# Complete genome sequence of *Citrobacter werkmanii* strain BF-6 isolated from industrial putrefaction

**DOI:** 10.1186/s12864-017-4157-9

**Published:** 2017-10-10

**Authors:** Gang Zhou, Hong Peng, Ying-si Wang, Xiao-mo Huang, Xiao-bao Xie, Qing-shan Shi

**Affiliations:** 1 Guangdong Institute of Microbiology, Guangzhou, Guangdong 510070 People’s Republic of China; 2 State Key Laboratory of Applied Microbiology Southern China, Guangzhou, Guangdong 510070 People’s Republic of China; 3 Guangdong Provincial Key Laboratory of Microbial Culture Collection and Application, Guangzhou, Guangdong 510070 People’s Republic of China; 4 Guangdong Open Laboratory of Applied Microbiology, Guangzhou, Guangdong 510070 People’s Republic of China

**Keywords:** *Citrobacter werkmanii*, Complete genome, Biofilm formation, RT-PCR, Evolutionary relationships

## Abstract

**Background:**

In our previous study, *Citrobacter werkmanii* BF-6 was isolated from an industrial spoilage sample and demonstrated an excellent ability to form biofilms, which could be affected by various environmental factors. However, the genome sequence of this organism has not been reported so far.

**Results:**

We report the complete genome sequence of *C. werkmanii* BF-6 together with the description of the genome features and its annotation. The size of the complete chromosome is 4,929,789 bp with an average coverage of 137×. The chromosome exhibits an average G + C content of 52.0%, and encodes 4570 protein coding genes, 84 tRNA genes, 25 rRNA operons, 3 microsatellite sequences and 34 minisatellite sequences. A previously unknown circular plasmid designated as pCW001 was also found with a length of 212,549 bp and a G + C content of 48.2%. 73.5%, 75.6% and 92.6% of the protein coding genes could be assigned to GO Ontology, KEGG Pathway, and COG (Clusters of Orthologous Groups) categories respectively. *C. werkmanii* BF-6 and *C. werkmanii* NRBC 105721 exhibited the closest evolutionary relationships based on 16S ribosomal RNA and core-pan genome assay. Furthermore, *C. werkmanii* BF-6 exhibits typical bacterial biofilm formation and development. In the RT-PCR experiments, we found that a great number of biofilm related genes, such as *bsmA*, *bssR*, *bssS*, *hmsP*, *tabA*, *csgA*, *csgB*, *csgC*, *csgD*, *csgE*, and *csgG*, were involved in *C. werkmanii* BF-6 biofilm formation.

**Conclusions:**

This is the first complete genome of *C. werkmanii*. Our work highlights the potential genetic mechanisms involved in biofilm formation and paves a way for further application of *C. werkmanii* in biofilms research.

**Electronic supplementary material:**

The online version of this article (10.1186/s12864-017-4157-9) contains supplementary material, which is available to authorized users.

## Background

The genus *Citrobacter* was introduced in 1932 by Werkman & Gillen and is a distinct group of aerobic, Gram-negative, non-spore-forming rod-shaped bacteria commonly found in water, soil, food and intestinal tracts of animals and humans [1, 2]. *Citrobacter* belongs to the family *Enterobacteriaceae* and some strains of this genus can cause serious opportunistic infections particularly involving the urinary and respiratory tracts [3–5]. In addition, *Citrobacter* sp. cause enteric diseases and may also be associated with extra-intestinal disorders, such as neonatal meningitis [6] and brain abscesses [7]. The species *Citrobacter werkmanii* was named to honor Chester H. Werkman, an American microbiologist, who studied the fermentative production of trimethylene glycol from glycerol and proposed the genus *Citrobacter* [1].

Based on their physiological properties, several *Citrobacter* sp. were used to deal with environmental pollution or produce biological metabolites. A great number of biofilm-immobilized *Citrobacter* sp. have been used for bioremediation of heavy metals via the activity of an acid-type phosphatase enzyme or their ability to accumulate heavy metals [8–10]. *C. werkmanii* DSM17579 was considered as a new candidate for the production of 1, 3-propanediol (PDO) using cheap waste streams such as ligno/hemicellulosic hydrolysates [11]. Through multiple knock-outs of the *dha* cluster encoding PDO producing enzymes, the concentration of the toxic intermediate 3-HPA in *Citrobacter* species was reduced to below detection limit and the maximal theoretical PDO yield on glycerol was reached [12, 13].

Microbial biofilms are defined as matrix-enclosed bacterial populations that adhere to each other and to biotic or abiotic surfaces [14]. It has been found that several gene clusters, such as the curli assembly protein cluster [15–17], contribute to bacterial biofilms formation and the above process can be influenced by multiple nutritional and environmental factors [18, 19]. In our previous study, a strain of *C. werkmanii* (named as BF-6) was successfully isolated from an industrial spoilage sample [20]. Our research indicated that the biofilms forming capacity of *C. werkmanii* BF-6 is affected by culture temperature, media, time, pH, and the osmotic agent glucose or sucrose. Confocal Laser Scanning Microscopy (CLSM) results illustrated that biofilms structure and extracellular polysaccharide of *C. werkmanii* BF-6 was influenced by NaCl or KCl in a concentration-dependent manner [21]. Additionally, we also found that denser biofilms were formed by *C. werkmanii* BF-6 in the presence of 400 mM Ca^2+^ when compared to 12.5 mM Ca^2+^ [22]. A total of 151 proteins from planktonic cells and biofilms were successfully identified after exposure of BF-6 cells to 12.5 and 400 mM Ca^2+^ and categorized into different gene ontology (GO) and KEGG pathways [22]. However, the definite functions of the altered proteins and their respective signal transduction pathways were elusive owing to the lack of *C. werkmanii* BF-6 genome information.

Up to now, there is only one submitted draft genome of *C. werkmanii* NBRC 105721 in the NCBI genome databases. Furthermore, the genomic structure and basic properties of *C. werkmanii* NBRC 105721 have not been reported. In this study, we sequenced the complete genome of *C. werkmanii* BF-6 and compared it with that of *C. werkmanii* NBRC 105721 and other strains of the genus *Citrobacter*.

## Results

### General genomic features

**Fig. 1:**
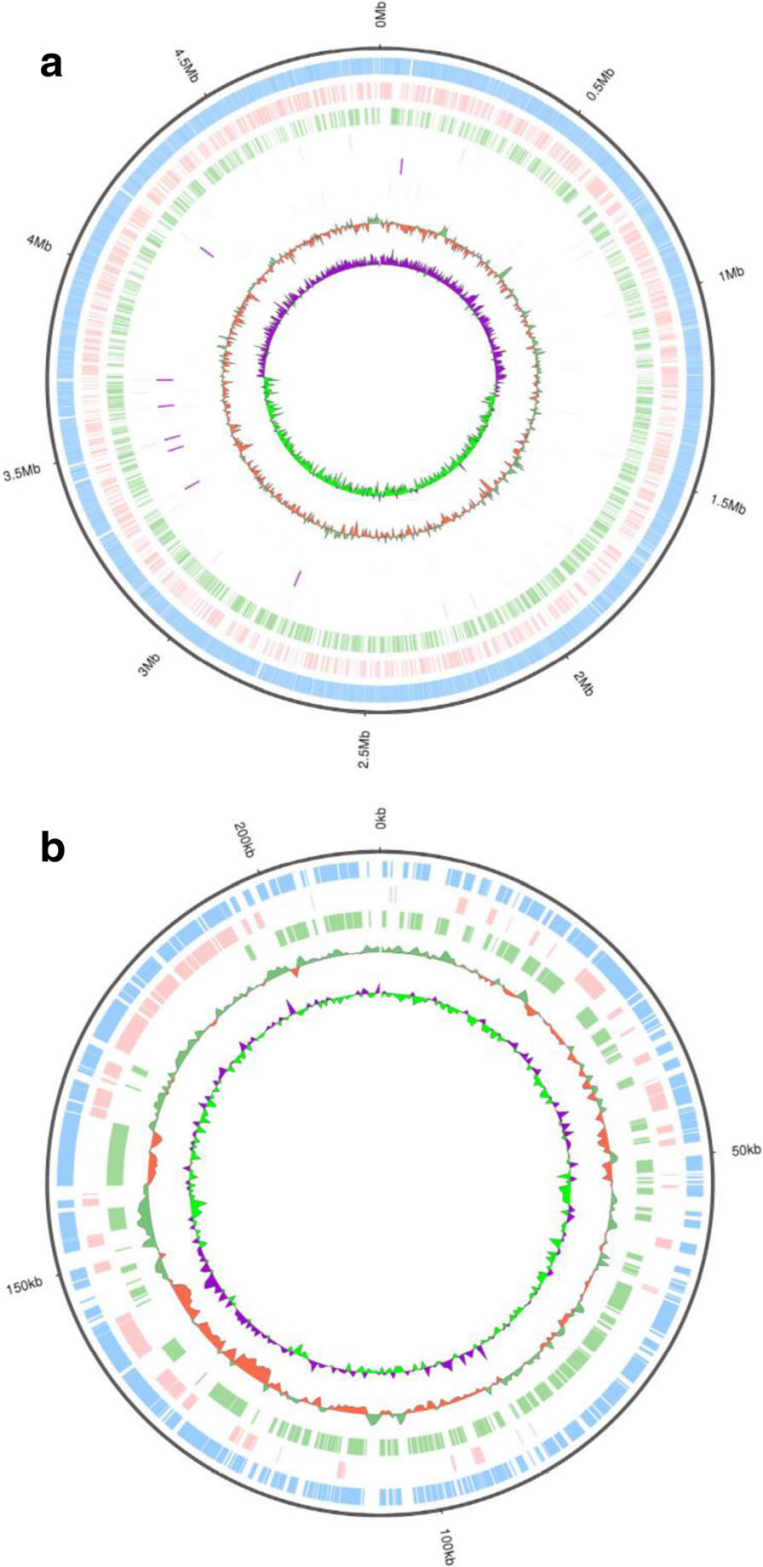
Circular representation of the complete genome of *C. werkmanii* BF-6 (**a**) and the plasmid pCW001 (**b**) displaying their relevant features, respectively. **a** From the inner- to the outermost circle: circle 1, GC skew (positive GC skew in green and negative GC skew in purple); circle 2, GC content; circle 3, sRNA; circle 4, rRNA; circle 5, tRNA; circle 6, genes on reverse strand; circle 7, genes on forward strand; circle 8, all annotated genes; circle 9, genome size. **b** The circular map of pCW001 was visualized in CGView. The features are the following from center to outside: GC skew, GC content, genes on reverse strand, genes on forward strand, all annotated genes, and plasmid size

The whole genome sequence of *C. werkmanii* BF-6 was obtained with no gaps by the Illumina Hiseq 4000 and Pacbio RSII platforms. The main features of the *C. werkmanii* BF-6 genome and the pCW001 plasmid are summarized in Table 1. The complete genome sequence of BF-6 comprises a 4,929,789 bp circular chromosome containing 4570 protein-coding genes, 3 microsatellite sequences, 34 minisatellite sequences, 25 rRNA genes and 84 tRNA genes with an average G + C content of 52.0% (Table 1; Fig. 1a). We also discovered a plasmid (named as pCW001) from BF-6 cells, which is 212,549 bp long with an average G + C content of 48.2% and contains 250 proteins including RepB family plasmid replication initiator protein, locus_tag: B2G73_RS24605, homologous protein No.: WP_007372338.1; plasmid stability protein, locus_tag: B2G73_RS25160, homologous protein No.: WP_009652914.1; plasmid stabilization protein, locus_tag: B2G73_RS25165, homologous protein No.: WP_007372199.1; etc. (Table 1; Fig. 1b).

**Table 1:** General features of the *C. werkmanii* BF-6 genome and the pCW001 plasmid

Items	Element and characteristics	Value
Chromosome	Size (bp)	4,929,789
	Proteins	4570
	Genes	4846
	G + C content (%)	52.0%
	tRNA genes	84
	rRNA operons	25
	Microsatellite sequences	3
	Minisatellite sequences	34
Plasmid *p*CW001	Size (bp)	212,549
	G + C content (%)	48.2%
	Proteins	250
	Genes	263

### Comparative genomics of *Citrobacter* sp. strains

**Fig. 2:**
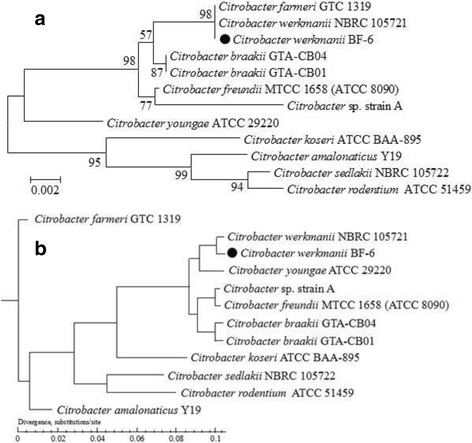
Dendrogram indicating the phylogenetic relationship of *C. werkmanii* BF-6 with other *Citrobacter* sp. **a** 16 s ribosomal RNA phylogeny. This figure was constructed using MEGA 6.0 based on the Neighbor-Joining method. Bootstrap values (expressed as percentages of 1000 replicates) are shown at branch points; (**b**) Whole-genome phylogeny based on core-pan genome analysis

To understand the evolutionary relationship of *C. werkmanii* BF-6 with other *Citrobacter* sp. strains, a 16S rRNA Neighbor-Joining phylogeny of BF-6 with 11 other *Citrobacter* sp. was performed. Phylogenetic analysis revealed close evolutionary relationship of *C. werkmanii* BF-6 with *C. werkmanii* NRBC 105721 (Fig. 2a). To further confirm this observation, a maximum-likelihood tree of the *C. werkmanii* BF-6 and 11 reported *Citrobacter* sp. complete genomes was also created based on core-pan genome analysis. These results also showed that *C. werkmanii* BF-6 is closely related to *C. werkmanii* NRBC 105721 (Fig. 2b). These results point towards a common evolutionary path between these two species of *Citrobacter*.

### Core and pan genes

**Fig. 3:**
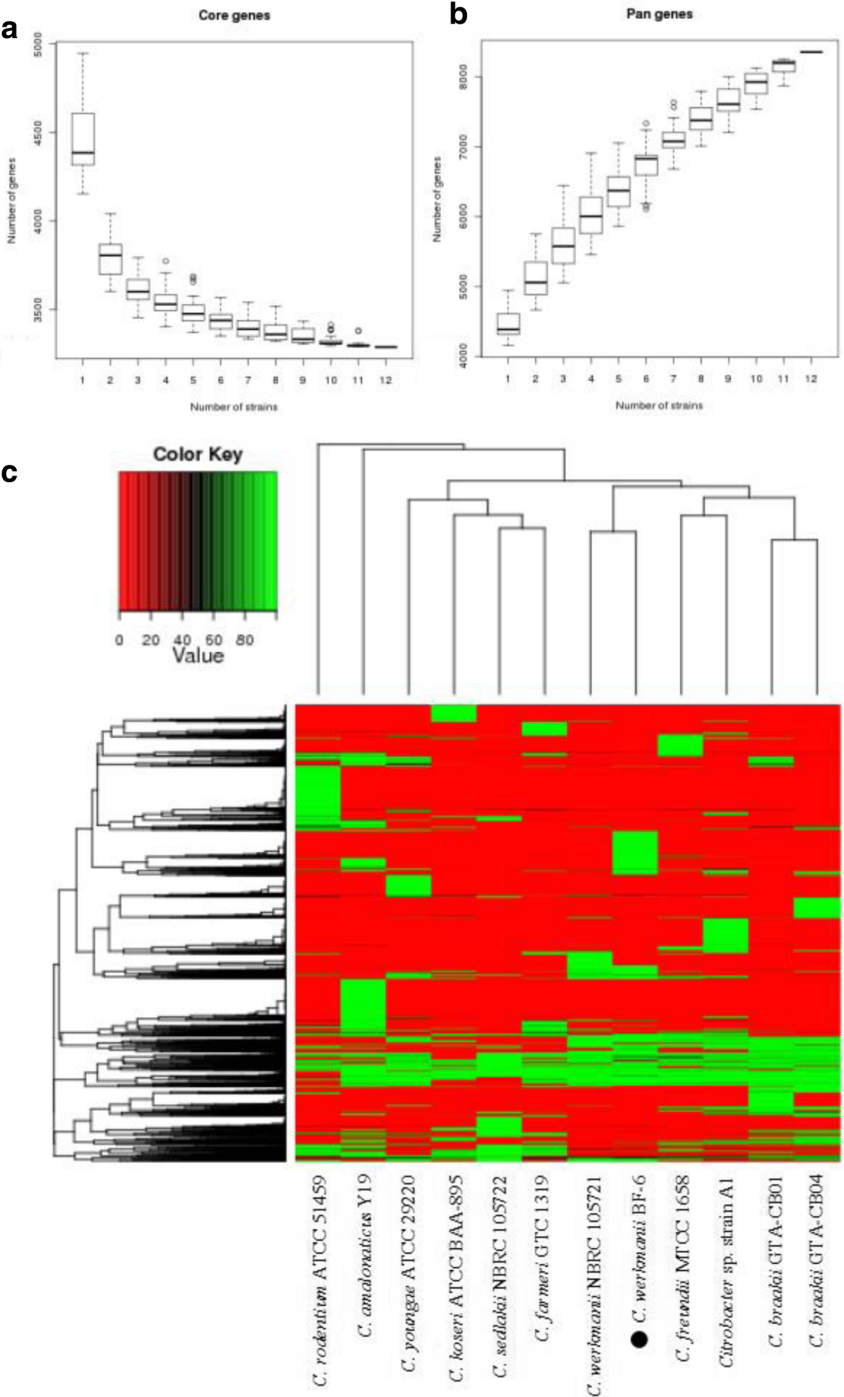
Analysis of the core and pan genome of *Citrobacter* sp. Dilution curve of bacterial core (**a**) and pan (**b**) genes. Heatmap was depicted after core gene deletion with the coverage cutoff >0.5 (**c**)

We compared the gene content of the *C. werkmanii* BF-6 genome with other *Citrobacter* sp. reference genomes using BLAST 2.2.26. The number of core genes decreased with the addition of new strains, whereas the pan genes continued to expand after addition of the 11 *Citrobacter* sp. genomes (Fig. 3a and b). The core *Citrobacter* genome contains 3450 genes, and the pan genome consists of 8356 genes shared among the 12 strains, including *C. werkmanii* BF-6. Detailed information about the core genes is listed in Additional file 1: Table S1. Many of the *C. werkmanii* BF-6 strain specific genes (239 and 30 genes located on the chromosome and plasmid pCW001, respectively) were hypothetical genes, the detailed information of which is provided in Additional file 2: Table S2. A heatmap after core gene deletion is also depicted (Fig. 3c). The above results demonstrated that *C. werkmanii* BF-6 and *C. werkmanii* NRBC 105721 shared the greatest similarity, providing molecular evidence for the similarities in phenotypes.

### Functional annotation of the *C. werkmanii* BF-6 genome

GO terms were assigned to *C. werkmanii* BF-6 genes for functional categorization. A total of 3361 genes were categorized into 42 subcategories belonging to the three main categories of biological process, cellular component and molecular function (Additional file 3: Figure S1). Among the 19 subcategories of biological process, most genes were assigned to cellular process, metabolic process and single organism process. In the cellular component category, a high percentage of genes belonged to cell, cell part, and membrane subcategories. Within the molecular function category, a majority of GO terms were grouped into catalytic activity, binding, and transporter activity subcategories. These GO annotations demonstrated that a wide variety of metabolic, structural, regulatory and transporter proteins were encoded by the *C. werkmanii* BF-6 genes.

To understand the intracellular metabolic pathways and functions of gene products, the genes were mapped to their corresponding terms in the KEGG pathway database. A total of 3453 genes were assigned to 40 KEGG pathways (Additional file 4: Figure S2). Carbohydrate metabolism (737, 21.3%) was the largest category, followed by signal transduction (533, 15.4%), overview (439, 12.7%), membrane transport (424, 12.3%), infectious disease (413, 12.0%), and amino acid metabolism (402, 11.6%). These functional annotations of the genes of *C. werkmanii* BF-6 provide a basis for exploring specific biological processes, functions, subcellular localization, and pathways of gene products in genome research.

The COG database classified gene products into different clusters of orthologous groups. In this study, 4234 genes of *C. werkmanii* BF-6 were classified into 4 first classes and 23 s classes of functional categories (Additional file 5: Figure S3). The top three categories were: carbohydrate transport and metabolism (474, 11.2%), amino acid transport and metabolism (433, 10.2%), and transcription (387, 9.1%). At the same time, the smallest group was RNA processing and modification (1, 0.02%).

### CLSM observation of BF-6 biofilms

**Fig. 4:**
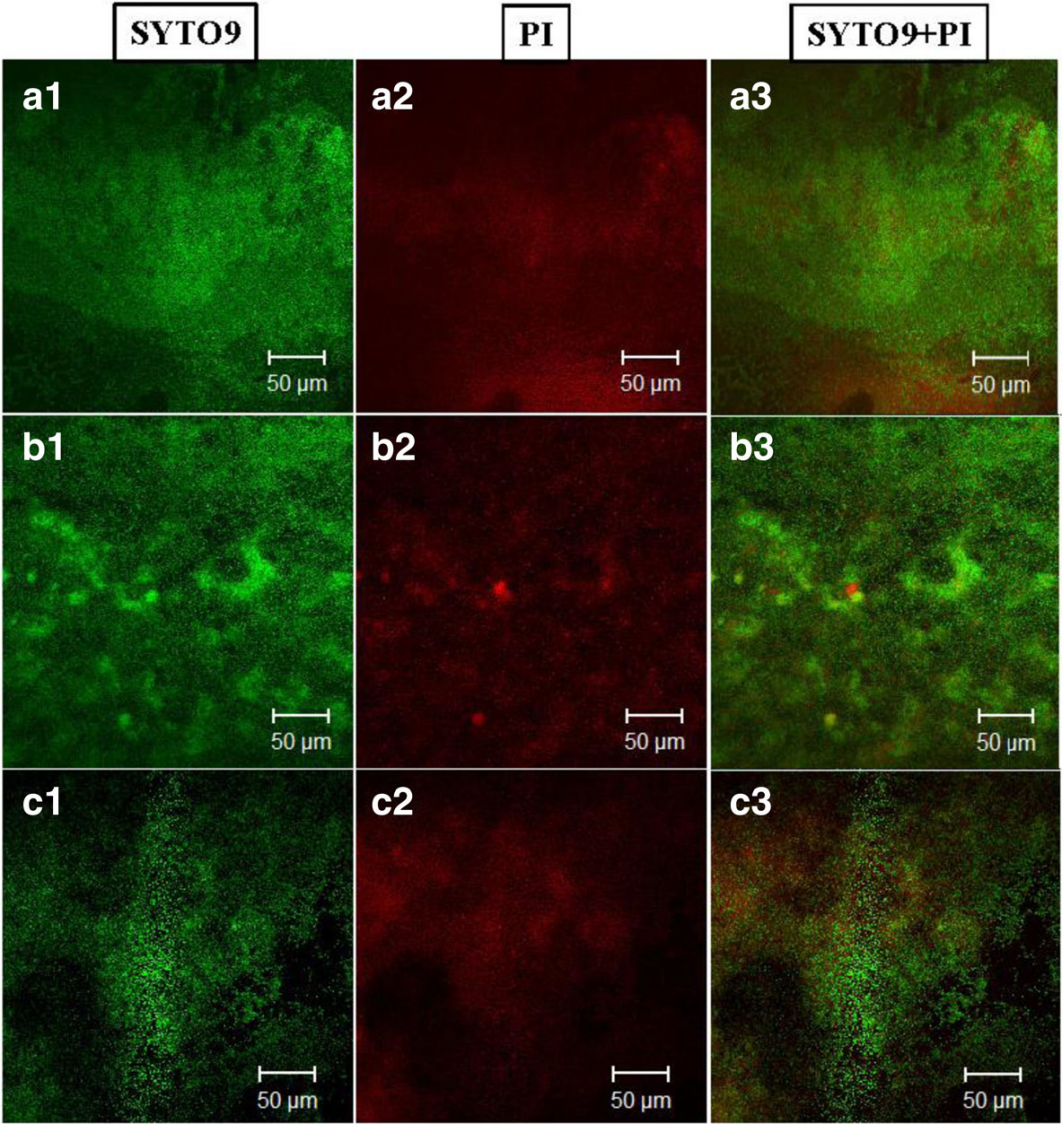
CLSM images of *C. werkmanii* BF-6 biofilms grown on glass slides and stained with SYTO9 and PI. A1, A2, A3: 2 days; B1, B2, B3: 4 days; C1, C2, C3: 6 days. Scale bar = 50 μm

In our previous study, we found that BF-6 possesses a high capacity for biofilm formation. Therefore the morphology, topography and architecture of BF-6 biofilms grown on glass cover slips over a period were observed using CLSM. As shown in Fig. 4, typical and denser biofilms were constructed by BF-6 on the second and fourth days, respectively. Biofilms dispersal was observed on the sixth day. Quantitative analysis of the BF-6 biofilms was conducted using COMSTAT 2.0 with at least 5 independent scans for each sample in the CLSM experiment. The highest values of total biomass, maximum and average thickness, were found on the fourth day (*p* < 0.05; Table 2). No differences in these values were observed on the second and sixth days (*p* > 0.05; Table 2). The results analyzed by COMSTAT 2.0 were thus inconsistent with the CLSM observations.

**Table 2:** Quantitation of biofilm architecture

Items	2 days	4 days	6 days
Total biomass (μm^3^/μm^2^)	19.86 ± 3.11^b^	24.84 ± 1.60^a^	19.67 ± 2.14^b^
Maximum thickness (μm)	9.20 ± 1.10^b^	13.20 ± 1.10^a^	8.40 ± 0.89^b^
Average thickness (μm)	7.00 ± 1.51^ab^	8.42 ± 1.55^a^	6.20 ± 1.38^b^

^*^Quantitation of biofilm parameters, including total biomass, maximum thickness, average thickness, were evaluated using COMSTAT. The results are means of datasets obtained from analysis of five CLSM images acquired at random positions in each of the biofilms. Standard deviations (SD) are also shown at the end of each of the means. Different superscript letters denote significant differences within a row (Tukey’s HSD: *P* < 0.05)

### Relative gene expression of selected biofilm formation genes

**Fig. 5:**
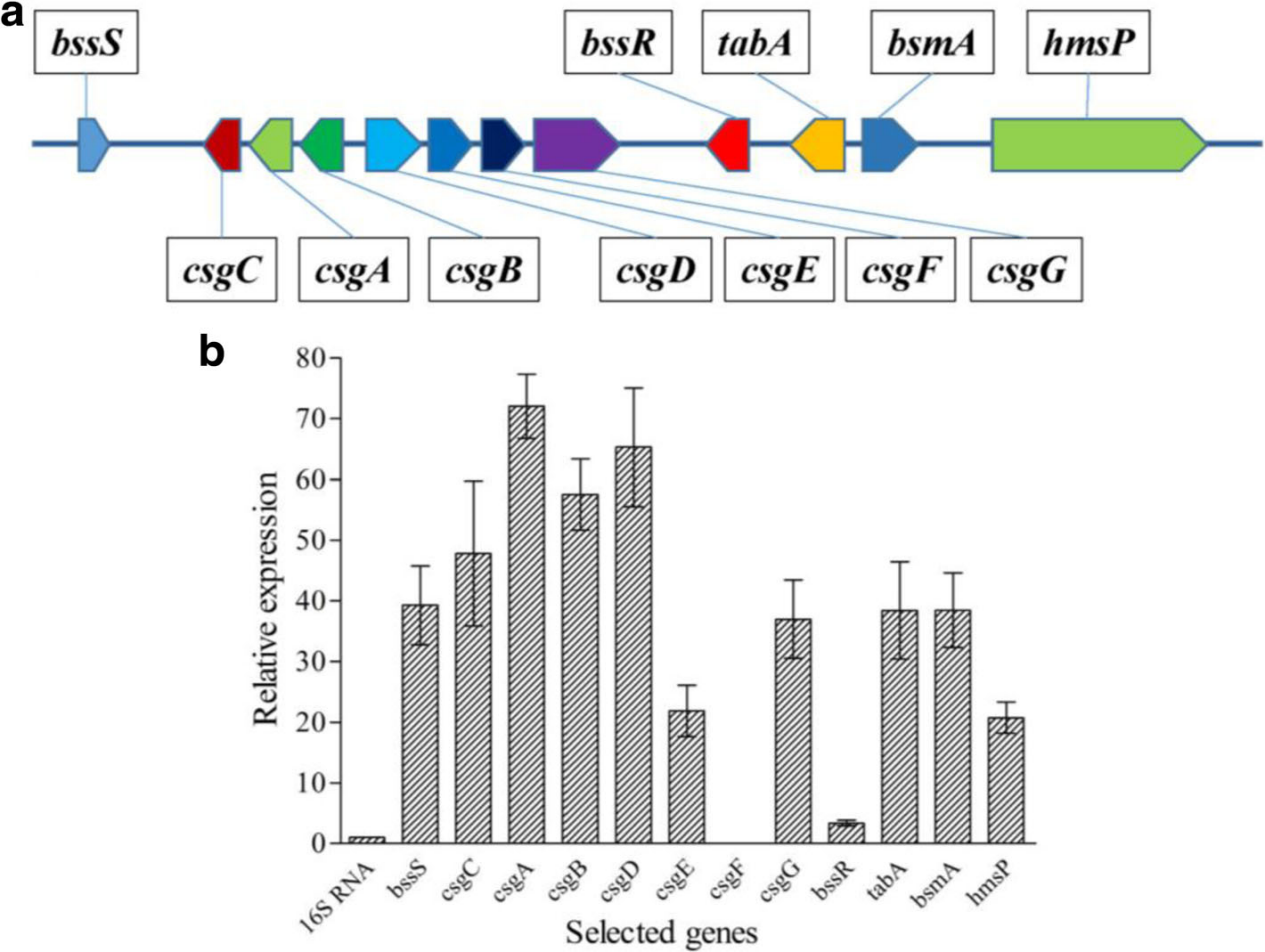
Schematic representation of the curli assembly protein gene cluster and other biofilm-formation related genes on the chromosome of *C. werkmanii* BF-6 (**a**), and their relative expression levels in the 2-day old biofilms as determined by qRT-PCR (**b**). All assays were performed in triplicate; mean values and standard deviations are shown

As reported in existing literature, several genes are involved in the process of biofilms formation [23, 24]. Therefore, partial genes related to biofilms formation in the genome of *C. werkmanii* BF-6 were selected and their expression levels in the planktonic cells and biofilms (2 days old) were calculated using RT-PCR (Additional file 6: Table S3). The 12 selected genes and their relative location sites on the chromosome of BF-6 are illustrated in the Fig. 5a. The relative expression levels of all tested genes except *csgF* were up-regulated, suggesting that they are involved in biofilms formation (Fig. 5b). Meanwhile, the changed expression trend of the selected biofilms formation genes on the fourth and sixth days was similar to that on the second day (data not shown).

## Discussion

In this study, the complete genome of *C. werkmanii* BF-6 was sequenced with no gaps and comparative genome analyses were also conducted within *Citrobacter* sp. To our knowledge, this is the first report of the complete genome map of *C. werkmanii*.

The genus *Citrobacter* is classified into 11 genomospecies based on DNA hybridization: *C. freundii*, *C. koseri*, *C. amalonaticus*, *C. farmeri*, *C. youngae*, *C. braakii*, *C. werkmanii*, *C. sedlakii*, *C. rodentium*, *C. gillenii*, and *C. murliniae* [25–27]. In our study, we found that the size of genome *C. werkmanii* BF-6 was 4,929,789 bp which is similar to that of *C. werkmanii* NBRC 105721 (4,947,997 bp) and *C. farmeri* GTC 1319 (4,929,495 bp, Table 3). Phylogenetic analysis revealed close evolutionary relationship of BF-6 with *C. farmeri* GTC 1319 and *C. werkmanii* NRBC 105721 based on 16S ribosomal RNA analyses. However, *C. werkmanii* BF-6 and *C. werkmanii* NRBC 105721 shared the closest evolutionary relationship according to a core-pan genome comparison (Fig. 1). Although *C. werkmanii* BF-6 and *C. werkmanii* NRBC 105721 have the closest evolutionary relationship, BF-6 has a plasmid of pCW001 that is not found in NRBC 105721 (Table 1; Fig. 2). Further research is needed to determine the function of the plasmid.

**Table 3:** General features of *Citrobacter* sp. strains

Organisms	Accession number	Size (Mb)	GC content (%)	Number of genes	tRNA	rRNA	Reference^*^
*Citrobacter* sp. strain A1	AKTT00000000	5,096,012	51.8%	4836	63	4	[57]
*Citrobacter freundii* MTCC 1658	ANAV00000000	5,001,265	51.6%	4691	70	10	[58]
*Citrobacter rodentium* DBS100 (ATCC 51459)	JXUN00000000	5,385,810	54.7%	5268	80	38	[59]
*Citrobacter youngae* ATCC 29220	NZ_ABWL00000000.2	5,150,259	52.7%	4950	79	14	–
*Citrobacter koseri* ATCC BAA-895	NC_009792	4,720,462	53.8%	4515	83	22	–
*Citrobacter braaki*i GTA-CB01	JRHK00000000	5,234,166	51.9%	4954	85	22	[60]
*Citrobacter braakii* GTA-CB04	JRHL00000000	5,036,963	52.0%	4698	23	23	[60]
*Citrobacter amalonaticus* Y19	NZ_CP011132	5,584,358	53.4%	5440	84	22	–
*Citrobacter sedlakii* NBRC 105722	NZ_BBNB01000001	4,631,466	54.7%	4463	74	6	–
*Citrobacter farmeri* GTC 1319	NZ_BBMX01000006	4,929,495	53.4%	4789	66	5	–
*Citrobacter werkmanii* NBRC 105721	NZ_BBMW00000000	4,947,997	52.1%	4860	68	5	–

“*”: the “-” indicate that the genomes of selected *Citrobacter* sp. strains have not been published

Meanwhile, general function analysis of the BF-6 genome demonstrated that the genes identified were primarily involved in carbohydrate transport and metabolism (Additional file 3: Figure S1, Additional file 4: Figure S2 and Additional file 5: Figure S3). Our previous study showed that glucose, mannitol, sorbitol, arabinose, but not inositol, sucrose and melibiose, can be used as carbon sources for growth [20]. In addition, we also found that both planktonic growth and biofilm formation of *C. werkmanii* BF-6 reduced with increasing concentration of glucose. At higher concentrations of glucose (800 and 1600 mM), most of the planktonic and biofilm growth was repressed [21]. These results demonstrated that glucose can be used by *C. werkmanii* BF-6 as a carbon source to grow or form biofilms only at lower concentrations, whereas higher concentrations may cause osmotic stress and inhibit growth. In *Escherichia coli*, the repressive effect of glucose is exerted through catabolite repression via the cAMP-CRP system [28]. Enzyme II A (EIIA) plays a central role in this system and there are different catabolite-specific EIIAs in a single cell [29]. In the *C. werkmanii* BF-6 genome, a great number of specificities of EIIA enzymes to different sets of catabolites were also found, such as PTS fructose transporter subunit IIC (Accession No.: WP_042306972.1), PTS fructose transporter subunit IIBC (WP_079223421.1), PTS mannose transporter subunit IID (WP_003833769.1), PTS mannose/fructose/sorbose transporter subunit IIC (WP_005122174.1), PTS glucose transporter subunit IIA and IIBC (WP_042312726.1 and WP_003036277.1), PTS trehalose transporter subunit IIBC (WP_042312710.1). Hence, we propose that carbon catabolite repression (CCR), a regulatory phenomenon by which the expression of genes for the use of secondary carbon sources and the activities of the corresponding enzymes is reduced in the presence of a preferred carbon source, also exists in *C. werkmanii* BF-6.

Typical biofilm development involves five stages: initial attachment of cells to the surface, production of EPS resulting in more firmly adhered “irreversible” attachment, early development of biofilm architecture, maturation of biofilm architecture, and dispersion of single cells from the biofilm [30]. From the CLSM images (Fig. 4) and biofilm architecture data (Table 3), we conclude that *C. werkmanii* BF-6 has a typical biofilm development process and structure. A large number of genes are involved in biofilms formation and development [31, 32]. It has been reported that *bsmA*, a quorum-sensing-regulated gene, is engaged in fine-tuning the formation of cell aggregates at a specific point in biofilm formation and development [33, 34]. Another pair of genes, *bssR* and *bssS*, appear to be global regulators of the uptake and export of signaling pathways, including quorum sensing [35]. Quorum sensing controls biofilm formation through modulation of Cyclic di-GMP levels, a signaling molecule that governs the transition between planktonic and biofilm states [36, 37]. HmsP, a putative phosphodiesterase, control Hms-dependent biofilm formation: a critical residue (E506) of HmsP within the EAL domain that is required for inhibition of biofilm formation is also essential for its phosphodiesterase activity in *Yersinia pestis* [38, 39]. Overexpression of YjgK (TabA), a component of the Toxin-Antitoxin system, decreased biofilm formation at 8 h and increased biofilm formation at 24 h; as expected deletion of *yjgK* also affected biofilm formation in *E. coli* by increasing biofilm formation after 8 h and decreasing biofilm formation after 24 h [40]. In this study, we found that the relative expression levels of *bsmA*, *bssR*, *bssS*, *hmsP*, and *tabA* were increased in the two-day old biofilms of *C. werkmanii* BF-6 (Fig. 5), suggesting that these genes are involved in biofilms formation. Curli assembly is guided by the products of seven curlispecific genes (*csg*) encoded on two divergently transcribed operons, *csgDEFG* and *csgBAC*. *csgD* is the master regulator of curli biogenesis and is required for transcription of the *csgBAC* operon [41]. Meanwhile, initial steps of biofilm development require transcription of genes involved in reversible attachment and motility, while the subsequent steps require genes involved in the irreversible attachment of bacteria [42]. In addition, the second irreversible step might require the synthesis of adhesive organelles, such as the curli fibers (*csg* genes) [43]. In this study, all genes of *csg* cluster except *csgF* were found to be up-regulated upon RT-PCR analysis (Fig. 5). *csgF* associates with the outer membrane and is required for cell association of the minor curli fiber subunit *csgB*. The miss detection of *csgF* may be due to the limitations of detection sensitivity of RT-PCR used in this study.

## Conclusions

In this study, the first complete genome of *C. werkmanii* was sequenced and reported. We found that the size of the complete chromosome of *C. werkmanii* BF-6 is 4,929,789 bp with an average G + C content of 52.0%, and encodes 4570 protein coding genes. Meanwhile, a previously unknown circular plasmid designed as pCW001 was also found with a length of 212,549 bp and a G + C content of 48.2%. Based on 16S ribosomal RNA and core-pan genome assay, we found that *C. werkmanii* BF-6 and *C. werkmanii* NRBC 105721 exhibited the closest evolutionary relationships. Furthermore, CLSM observation showed that *C. werkmanii* BF-6 possessed a typical biofilm developing capability and exhibited a high and stable biofilms. In the RT-PCR experiments, we also found that a great number of biofilm related genes, such as *bsmA*, *bssR*, *bssS*, *hmsP*, *tabA*, *csg* gene cluster, were involved in *C. werkmanii* BF-6 biofilm formation. Overall, the elucidation of the complete genome of *C. werkmanii* BF-6 provides a stable molecular foundation for genetic modification and industrial utilization of this strain.

## Methods

### Bacterial strain and culture conditions

*C. werkmanii* BF-6 was previously isolated from an industrial putrefaction sample and has been deposited at the Guangdong Culture Collection Center (Guangzhou, Guangdong, China) under the accession number GDMCC 1.1242. BF-6 was routinely inoculated into Luria Bertani (LB) medium and cultured at 30 °C and 165 rpm for the required time in a shaker incubator. All chemicals used in this study were reagent grade and purchased from Sigma (St Louis, MO, USA) unless indicated otherwise.

### Whole-genome sequencing

Cells of *C. werkmanii* BF-6 were harvested in exponential phase at an optical density of 0.6 at 600 nm. The genomic DNA of BF-6 was extracted using QIAamp DNA Mini Kit (Qiagen, CA, USA) according to the manufacturer’s protocol and fragmented randomly through Covaris or Bioruptor method. After fragmentation, the overhangs of the short-fragments were converted into blunt ends using T4 DNA polymerase (NEB, Beijing, China), Klenow DNA Polymerase (NEB) and T4 PNK (NEB). Sequencing adaptors were ligated to their 3′ ends and target segments that met the required length were isolated by gel electrophoresis after PCR amplification. The prepared libraries were sequenced using Illumina Hiseq 4000. To construct the complete genome of BF-6, the extracted genome was also sequenced by the Pacbio RSII platform. Briefly, library preparation (SMRTbell) was performed according to the manufacturer’s protocols (Pacific Biosciences, Menlo Park, CA, USA). The prepared SMRTbell library was quantified using Qubit 2.0 Fluorometer (Invitrogen, Shanghai, China).

### Genome assembly and annotation

For quality control of the sequencing data, clean reads were obtained by removing reads with low quality, mismatched reads and duplicated reads using PreAssembler Filter v1 of SMRT analysis [44]. De novo assembly was performed with the help of SOAPdenovo 2.04 [45, 46], SMRT Analysis software v2.2.0 (Pacific Biosciences) featuring HGAP 2 [44], with subsequent correction by quiver in addition to Gepard v1.30 [47]. Putative open reading frames (ORFs) were predicted using Glimmer 3.02 [48] and GeneMark.hmm [49], and putative ORF functions were analyzed by BLASTP (Coverage ≥40% and identity >40%) and InterProScan [50]. All ORFs were also translated and aligned using the NCBI non-redundant database, SwissProt database, Gene Ontology (GO), Clusters of Orthologous Groups (COG) and Kyoto Encyclopedia of Genes and Genomes (KEGG).

### Comparative study on the core and pan genome

The comparative study on the core and pan genome was conducted according to the previlusly reported methods [51, 52]. Briefly, the gene set in *Citrobacter sedlakii* NBRC 105722 was selected and regarded as the Reference and the gene sets in the other 11 *Citrobacter* sp. genomes were considered as the Query. The Query genes in each genome were aligned against the Reference genes in reference strain using BLAST v2.2.26 (http://blast.ncbi.nlm.nih.gov/Blast.cgi) and the blast results were filtered by their length and identity. Each gene in the Reference and Query gene sets was calculated with the BLAST Coverage Ratio (BCR) using the following formula: BCR (Reference) = (Match/Length (Reference)) × 100% and BCR (Query) = (Match/Length (Query) × 100%. Aligned by the genes from samples, the BCR values of genes from pan gene pool were calculated for each sample, and then the coverage array was generated for the pan gene pool. If the BCR value of the gene was larger than the setting value in each sample, the gene was the core gene. If the gene was predicted from assemble result, the blast results should be filtered, and the sequence should be removed if the number of N was large than the setting (30% as fault setting) in the gene.

### Phylogenetic analysis and genomic comparison

To understand the evolutional relationship between *C. werkmanii* BF-6 and 11 *Citrobacter* sp. strains, a phylogenetic tree was constructed using MEGA6 according to their 16S ribosomal RNA. For comparative genomic analysis of the BF-6 strain, genome sequences of these 11 *Citrobacter* sp. strains (Table 2) were downloaded from NCBI. The subprogram phyml of TreeBeST (http://treesoft.sourceforge.net/treebest.shtml) was used to construct a phylogenetic tree with the default parameters. Non-parametric bootstrap analysis with thousand re-samplings was conducted to obtain bootstrap values for all branches.

### Confocal laser scanning microscopy for analysis of biofilms

Based on our preliminary analysis, we found that BF-6 possessed a high capability for bacterial biofilm formation. To detect biofilm formation development, we observed the change of morphology and three-dimensional structures over a period using Confocal Laser Scanning Microscopy (CLSM) according to previously described methods [22, 53]. Briefly, a portion of coverslip was placed in a 24-well microtiter plate inoculated with aliquots of 2 ml BF-6 overnight cultured supernatant with an optical density of 0.05. The microtiter plates were then placed into a static incubator at 30 °C for 2, 4 or 6 days. On the indicated day, the glass slides were gently taken out from microtiter plates and washed gently with deionized water to remove loosely attached planktonic cells. The attached biofilms on the coverslip was stained with 5 μM SYTO9 fluorescent dye (Invitrogen, Carlsbad, CA, USA) and 30 μM propidium iodide (Sigma Chemical Co., St. Louis, MO, USA) for at least 15 min. Subsequently, the slides were washed gently again with deionized water and the stained biofilms were visualized using CLSM (LSM 710 Zeiss, Jena, Germany). Finally, quantification of biofilm structures was evaluated using COMSAT 2.0 software based on the obtained serial CLSM figures [54, 55].

### RT-PCR

The planktonic cells and biofilms of *C. werkmanii* BF-6 were cultured in 24-well microtiter plates as described in the CLSM assay section above. On the indicated days, the planktonic bacteria were pipetted into a new plastic tube and the attached biofilms on the slides were also scraped into a new tube, respectively. After washing with diethylpyrocarbonate-treated water (DEPC-water) and centrifugation (10,000 rpm × 5 min) for three times, total RNAs of the collected samples were isolated using EasyPure RNA Kit (TransGen Biotech, Beijing, China) and converted into cDNA using the TransScript One-Step gDNA Removal and cDNA Synthesis SuperMix (TransGen) according to the manufacturer’s instructions. Ten-fold dilutions of the cDNA samples were used as templates for assessing the transcriptional levels of the 12 selected biofilms formation related genes via qRT-PCR with paired primers and 16S rRNA as an internal standard and sterile deionized water as the negative control (Additional file 6: Table S3). All qRT-PCRs were performed on a Mastercycler ep realplex (Eppendorf, Hamburg, Germany) with TransStart Top Green qPCR SuperMix (TransGen) following the manufacturer’s guides. The transcription level of each gene in the cDNA preparation was assessed using the 2^-ΔΔCt^ method [56]. The ratio of the transcription level of a given biofilms formation gene from BF-6 biofilms or planktonic cells grown at the second, fourth or sixth days to that from the planktonic BF-6 collected at the second day was defined as the relative transcription level.

## Additional files

Additional file 1: Table S1. Core genes of 12 *Citrobacter* sp. (XLS 3788 kb)

Additional file 2: Table S2. *C. werkmanii* BF-6 strain specific genes. (XLS 52 kb)

Additional file 3: Figure S1. Gene ontology (GO) analysis of *C. werkmanii* BF-6 genome. GO analysis of *C. werkmanii* BF-6 genome based on GO second level terms, corresponding to 3361 genes for their predicted involvement in biological process (blue), cellular component (brown) and molecular function (yellow). Classified gene objects are depicted as gene numbers. (DOCX 44 kb)

Additional file 4: Figure S2. KEGG pathway classifications of genes encoded by the *C. werkmanii* BF-6 genome based on the KEGG database. Functional classifications were assigned to a total of 3453 genes, and the numbers with each classification are indicated. (DOCX 111 kb)

Additional file 5: Figure S3. Functional classification of genes encoded by *C. werkmanii* BF-6 genome based on the COG database. A total of 4234 genes with orthologs in the COG database were classified and the numbers with each classification are indicated. (DOCX 44 kb)

Additional file 6: Table S3. Primers used for amplification of biofilm genes in the qRT-PCR assay. (DOCX 13 kb)

## Abbreviations

CLSM: Confocal Laser Scanning Microscopy
COG: Cluster of Orthologous Groups of Proteins
EPS: Extracellular polymeric substance; SD: Standard deviations
GO: Gene Ontology
KEGG: Kyoto Encyclopedia of Genes and Genomes
PDO: 1, 3-propanediol
