# Supplementary material for: Complete genome sequence of Citrobacter werkmanii strain BF-6 isolated from industrial putrefaction
Source: BMC Genomics. 2017 Oct 10;18:765. doi: 10.1186/s12864-017-4157-9 (PMC5635574; doi:10.1186/s12864-017-4157-9)
Supplement: Supplementary file 3 — Gene ontology (GO) analysis of C. werkmanii BF-6 genome. GO analysis of C. werkmanii BF-6 genome based on GO second level terms, corresponding to 3361 genes for their predicted involvement in biological process (blue), cellular component (brown) and molecular function (yellow). Classified gene objects are depicted as gene numbers. (DOCX 44 kb) [file 12864_2017_4157_MOESM3_ESM.docx]

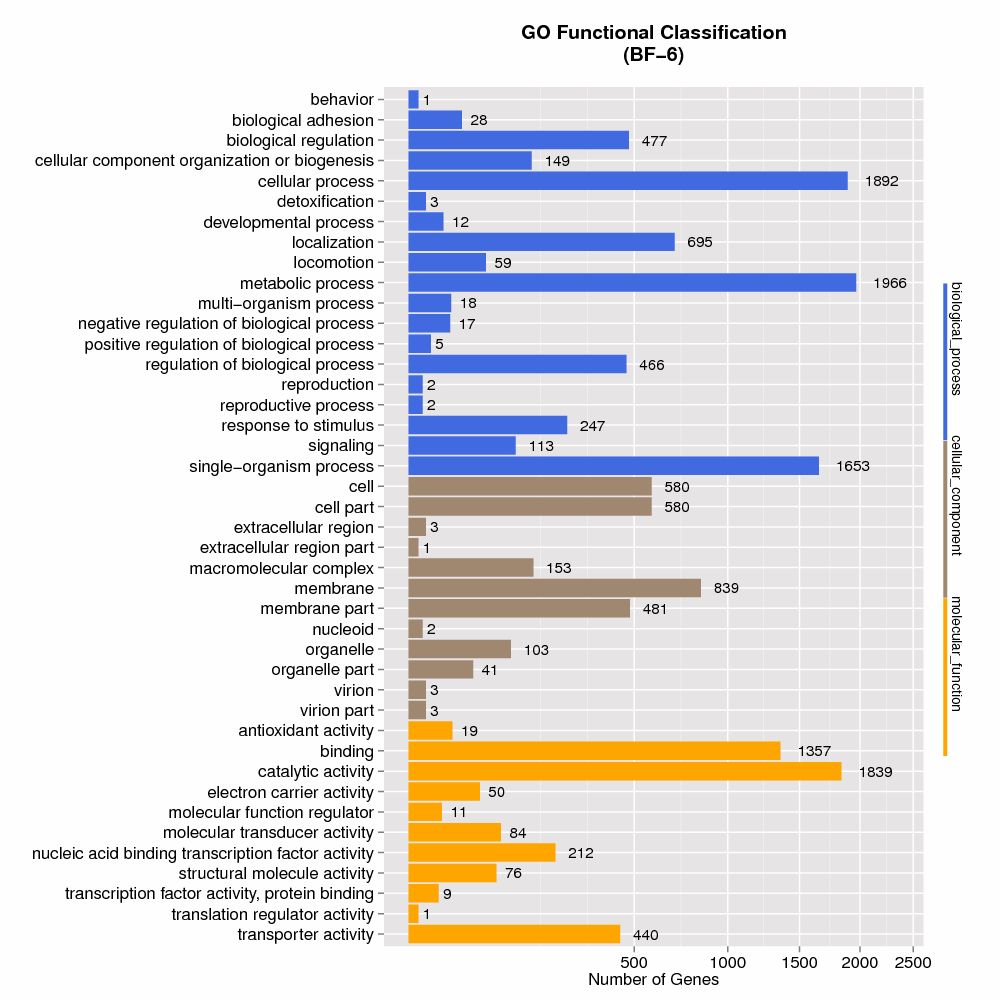


**Figure S1.** Gene ontology (GO) analysis of *C. werkmanii* BF-6 genome. GO analysis of *C. werkmanii* BF-6 genome based on GO second level terms, corresponding to 3,361 genes for their predicted involvement in biological process (blue), cellular component (brown) and molecular function (yellow). Classified gene objects are depicted as gene numbers.
