# Supplementary material for: Complete genome sequence of Citrobacter werkmanii strain BF-6 isolated from industrial putrefaction
Source: BMC Genomics. 2017 Oct 10;18:765. doi: 10.1186/s12864-017-4157-9 (PMC5635574; doi:10.1186/s12864-017-4157-9)
Supplement: Supplementary file 4 — KEGG pathway classifications of genes encoded by the C. werkmanii BF-6 genome based on the KEGG database. Functional classifications were assigned to a total of 3453 genes, and the numbers with each classification are indicated. (DOCX 111 kb) [file 12864_2017_4157_MOESM4_ESM.docx]

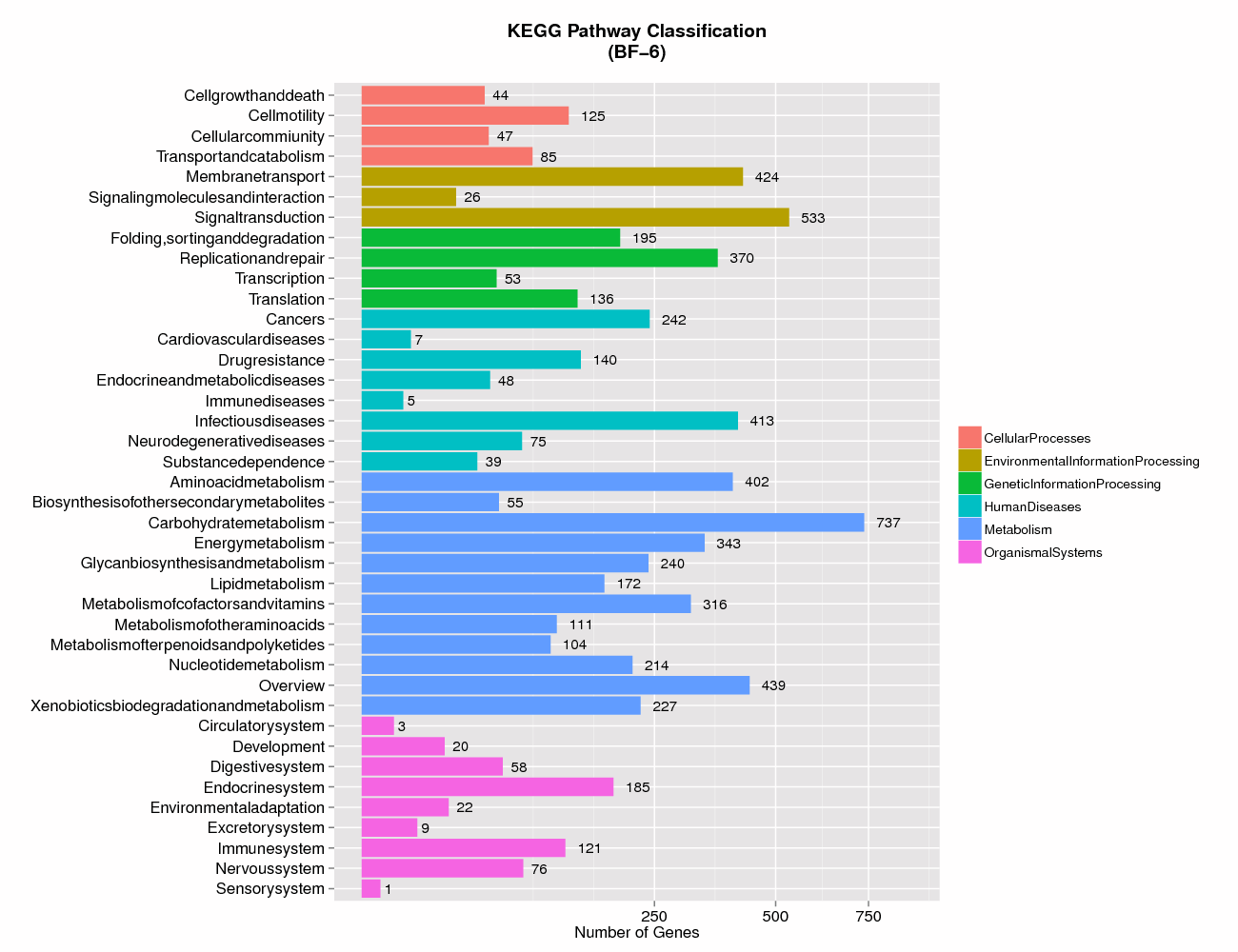


**Figure S2.** KEGG pathway classifications of genes encoded by the *C. werkmanii* BF-6 genome based on the KEGG database. Functional classifications were assigned to a total of 3,453 genes, and the numbers with each classification are indicated.
