# Supplementary material for: Complete genome sequence of Citrobacter werkmanii strain BF-6 isolated from industrial putrefaction
Source: BMC Genomics. 2017 Oct 10;18:765. doi: 10.1186/s12864-017-4157-9 (PMC5635574; doi:10.1186/s12864-017-4157-9)
Supplement: Supplementary file 5 — Functional classification of genes encoded by C. werkmanii BF-6 genome based on the COG database. A total of 4234 genes with orthologs in the COG database were classified and the numbers with each classification are indicated. (DOCX 44 kb) [file 12864_2017_4157_MOESM5_ESM.docx]

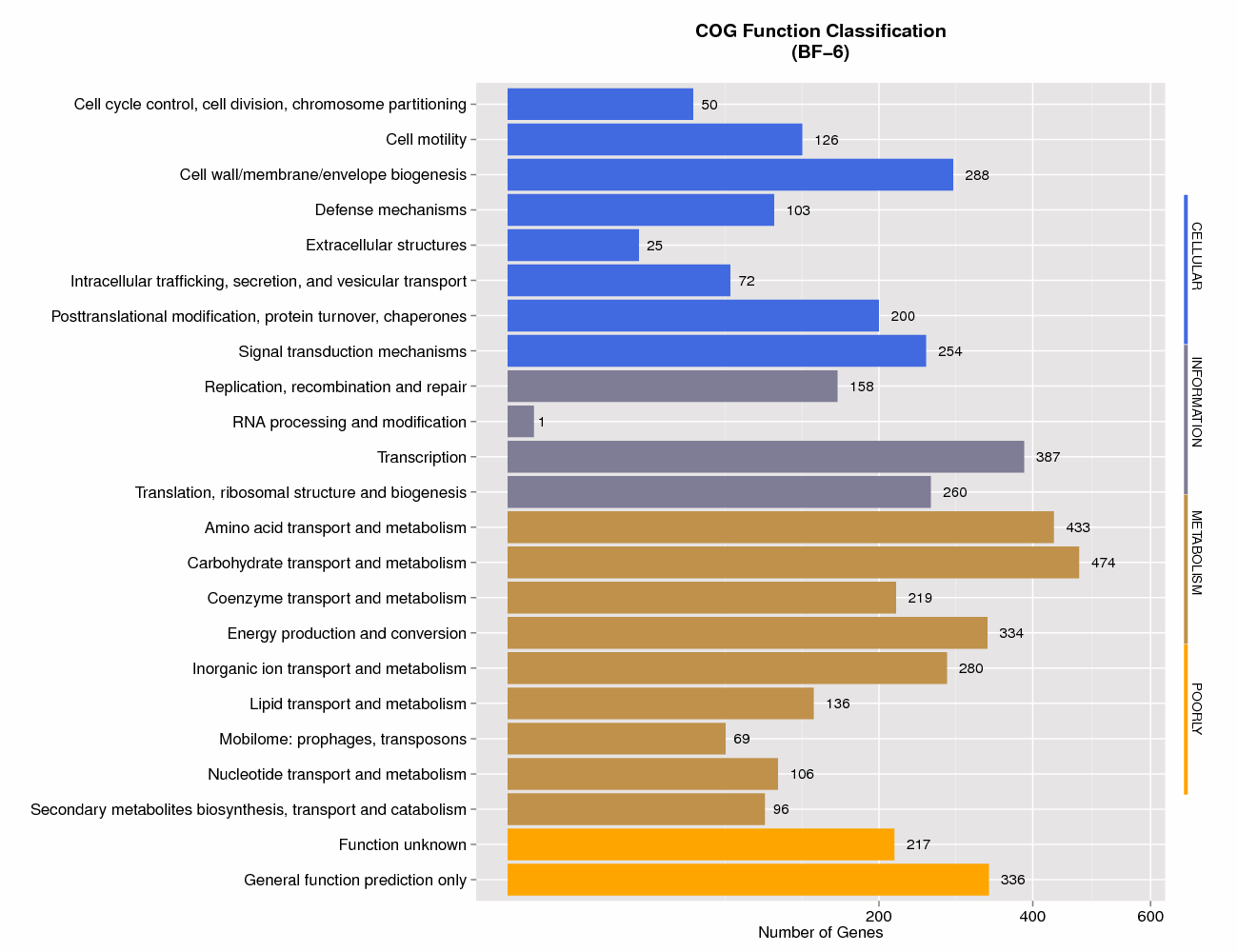


**Figure S3.** Functional classification of genes encoded by *C. werkmanii* BF-6 genome based on the COG database. A total of 4,234 genes with orthologs in the COG database were classified and the numbers with each classification are indicated.
