# Supplementary material for: Complete genome sequence of Citrobacter werkmanii strain BF-6 isolated from industrial putrefaction
Source: BMC Genomics. 2017 Oct 10;18:765. doi: 10.1186/s12864-017-4157-9 (PMC5635574; doi:10.1186/s12864-017-4157-9)
Supplement: Supplementary file 6 — Primers used for amplification of biofilm genes in the qRT-PCR assay. (DOCX 13 kb) [file 12864_2017_4157_MOESM6_ESM.docx]

**Table 3** Primers used for amplification of biofilm formation related genes in qRT-PCR assay

| Accession number or locus tag | Gene names | Annotation | Primer sequence (5'- 3') |
| --- | --- | --- | --- |
| KC489166 | 16S RNA | 16S ribosomal RNA | Forward: TTACCTACTCTTGACATC |
|  |  |  | Reverse: GACTTAACCCAACATTTC |
| B2G73_RS15900 | *bsmA* | biofilm peroxide resistance protein | Forward: TAATGGGTTACAGCGAATAG |
|  |  |  | Reverse: ATAAGACCACATAATAATCAGC |
| B2G73_RS10300 | *bssR* | biofilm formation regulatory protein | Forward: CGCTTATCTGCTGTTGAG |
|  |  |  | Reverse: ATACCGTGAAGTTGTGATTG |
| B2G73_RS09175 | *bssS* | biofilm formation regulatory protein | Forward: GGACTGAAGTTGGACAAA |
|  |  |  | Reverse: CGCTGATACTCATTTACCT |
| B2G73_RS19460 | *hmsP* | biofilm formation regulator | Forward: GTTAATACTCACGGTAGC |
|  |  |  | Reverse: GGTAATGCCAGTTGATAG |
| B2G73_RS15490 | *tabA* | toxin-antitoxin biofilm protein | Forward: GTCGGCAATATTCACAAC |
|  |  |  | Reverse: TCATATCTTCGGCAATCA |
| B2G73_RS09265 | *csgA* | major curli subunit | Forward: GATTCTTCAATGAGCATCTAC |
|  |  |  | Reverse: GTCTGTTTCAGGTTAATGGTA |
| B2G73_RS09270 | *csgB* | curli subunit | Forward: AGACGGGATCATATAACTTT |
|  |  |  | Reverse: CTTTATTACCAGAGCCTTTC |
| B2G73_RS09260 | *csgC* | curli assembly protein | Forward: CGATTGAACTAACTAAACTGAG |
|  |  |  | Reverse: TTATGGCTGACTGGTTGA |
| B2G73_RS09280 | *csgD* | transcriptional activator of curli operon | Forward: GCGTTATTACAGCACTTA |
|  |  |  | Reverse: TTATCTGCCTCCATCATAT |
| B2G73_RS09285 | *csgE* | curli assembly protein | Forward: CGTTATCTTCCAGACCTT |
|  |  |  | Reverse: GCTTGTGCTTAATAGTGTT |
| B2G73_RS09290 | *csgF* | curli assembly protein | Forward: CAGAACTCGTATAAAGAT |
|  |  |  | Reverse: CGGTATTGATATTAGTGA |
| B2G73_RS09295 | *csgG* | curli assembly protein | Forward: TTATACCTCTAACGAACC |
|  |  |  | Reverse: GATGTCATTATCAACTTGT |
